# Paracostal versus ventral midline approach for caudate liver lobectomy in the rabbit

**DOI:** 10.1111/vsu.13838

**Published:** 2022-06-13

**Authors:** Katherine C. Leonard, Qianqian Zhao, Rachel H. Taber, Sara A. Colopy

**Affiliations:** ^1^ Department of Surgical Sciences, School of Veterinary Medicine University of Wisconsin‐Madison Madison Wisconsin USA; ^2^ Department of Biostatistics and Medical Informatics University of Wisconsin – Madison Madison Wisconsin USA

## Abstract

**Objective:**

To describe the paracostal approach to caudate liver lobectomy in rabbits and compare the outcome of paracostal versus ventral midline approach for caudate liver lobectomy in rabbits with caudate liver lobe torsion (LLT).

**Study design:**

Cadaveric and retrospective study.

**Animals:**

Normal rabbit cadavers (n = 5) and rabbits with caudate LLT (n = 22).

**Methods:**

Cadavers – a right paracostal or ventral midline approach was made. Accessibility of the caudate liver lobe and relationship to the gastrointestinal (GI) tract were assessed. Clinical LLT cases – 9 cases were treated via the paracostal approach and 13 were treated via the ventral midline approach. Medical records (January, 2018 to October, 2021) were reviewed. Anesthesia and surgical times, mortality rate, and relevant clinical data were compared between groups.

**Results:**

In cadavers, caudate liver lobectomy was feasible through a paracostal approach without retraction of the GI tract. In clinical cases, there was no difference in anesthesia time (*P* = 0.1397) or surgical time (*P* = 0.9462) between groups. All rabbits that underwent paracostal approach survived to discharge. Mortality was lower (*P* = .053) and postoperative time until eating was shorter (*P* = .0238) for patients undergoing paracostal approach.

**Conclusion:**

Rabbits experienced lower mortality and shorter time until eating when treated through a right paracostal approach compared to the ventral midline approach. The paracostal approach resulted in minimal to no manipulation of the GI tract.

**Clinical significance:**

A right paracostal approach for caudate liver lobectomy in rabbits provides good exposure while avoiding GI tract manipulation. This approach may result in improved survival and earlier eating in rabbits with caudate LLT.

## 1 INTRODUCTION

Liver lobe torsion (LLT) is a rare condition that has been reported in multiple species including humans, dogs, horses, pigs, otters, guinea pigs, lemurs, camels, and rabbits.^1^, ^2^, ^3^, ^4^, ^5^, ^6^, ^7^, ^8^, ^9^, ^10^, ^11^, ^12^, ^13^, ^14^, ^15^ Torsion of the left lateral liver lobe is most commonly reported in dogs; the caudate lobe is most commonly affected in rabbits.^2^ This is thought to be most likely due to its narrow dorsal attachment to the hilus of the liver.^16^

Clinical signs for LLT in rabbits can be nonspecific and similar to signs associated with gastrointestinal (GI) stasis. Prior to 2014, there were few reports of rabbit LLT in the literature. It has been suggested that LLT in rabbits is not a new disease but rather is becoming increasingly recognized as a cause of GI stasis in rabbits because advanced diagnostic imaging is more often being utilized.^2^ Although some rabbits may recover with medical management alone, prompt diagnosis and early surgical intervention is recommended.^1^, ^2^, ^6^ Lobectomy of the torsed liver lobe is considered the treatment of choice, and long‐term prognosis for patients surviving to discharge is reported to be excellent.^1^, ^2^, ^8^, ^10^ In a case series of 16 rabbits treated for LLT, 43% of the 7 rabbits survived when treated with supportive care, whereas all 9 rabbits treated surgically survived.^2^ Based on the 2014 study, liver lobectomy is currently recommended for all rabbits with LLT; however, not all rabbits survive surgery, in the authors' experience.

The GI tracts of rabbits can make access to the liver challenging. Further, excessive manipulation of the GI tract commonly results in GI stasis and/or adhesions.^17^ Intestinal and colonic obstruction secondary to postspay adhesions has been reported.^18^, ^19^ Adhesions associated with GI surgery can also occur in rabbits.^20^, ^21^ Minimal, gentle handling of the GI tract is therefore recommended to prevent occurrence of these postoperative complications.^22^

A ventral midline approach for liver lobectomy in rabbits has been recommended to allow access to all liver lobes.^1^, ^2^, ^4^, ^8^, ^10^, ^14^, ^22^ However, the stomach is commonly distended in rabbits with LLT secondary to GI stasis, and a ventral midline approach requires prolonged retraction of the stomach to access the hilus of the liver lobe. Given that the vast majority of rabbit LLTs occur in the caudate lobe, and the fact that computed tomography (CT) and ultrasound (US) are highly sensitive and specific for diagnosing LLT, careful preoperative planning may allow for a less invasive approach to liver lobectomy in rabbits.^23^

The purpose of this study was to describe a novel right paracostal approach for caudate liver lobectomy in rabbits. The second purpose was to compare clinically relevant data between rabbits undergoing caudate liver lobectomy via a right paracostal approach versus a traditional ventral midline approach. Our hypothesis was that this approach would be technically feasible, with shorter surgical times, lower mortality, and fewer intraoperative or postoperative complications.

## 2 MATERIALS AND METHODS

### 2.1 Cadaver study

Adult, mixed breed, rabbit cadavers (*n* = 5) obtained from rabbits euthanized for reasons unrelated to the study, were used. All rabbit cadavers were donated by Covance Laboratories, Inc., Madison, Wisconsin. Four cadavers were used to determine the feasibility of a right paracostal approach to the caudate liver lobe, and a single additional rabbit was used for a standard ventral midline approach for comparison. For rabbits in the paracostal group, the right lateral thorax and abdomen were clipped. A caudate liver lobectomy was performed through a right paracostal approach in all 4 rabbits as described below. The caudate liver lobe was identified and its relationship to the rib cage, GI tract, and right kidney were described. The caudate liver lobe was removed using encircling ligatures with 2‐0 silk suture.

### 2.2 Clinical cases

Medical records of rabbits diagnosed with caudate LLT at University of Wisconsin Veterinary Care between January 2018 and October 2021 were reviewed. A rabbit was included if it had caudate liver lobectomy performed for treatment of a caudate LLT through a ventral midline or right paracostal approach. Rabbits were not included in the study if a different liver lobe was affected (eg, the left lateral liver lobe) or if the liver lobe was removed for a reason other than LLT. Data recorded were date of surgery, age, breed, weight, sex, clinical signs, physical examination findings, preanesthetic bloodwork values, method of diagnostic imaging, American Society of Anesthesiologists (ASA) physical status, anesthetic and surgery duration, surgical approach, surgeon performing the technique, method of vessel ligation, intraoperative and postoperative complications, time until eating after surgery, hospitalization time after surgery, and perioperative mortality.

Intraoperative complications were defined as complications that occurred while the patient was anesthetized, and postoperative complications were defined as complications that occurred after recovery from general anesthesia and before discharge from the hospital. Based on complications recorded for each rabbit, the primary complications analyzed statistically were intraoperative hemorrhage, postoperative transfusion, and death.

### 2.3 Surgical technique

#### 2.3.1 Paracostal approach

Patients were positioned in left lateral recumbency. The patients' fur was clipped from the midthorax cranially to the midabdomen caudally, and from the dorsal midline to ventral midline. The area was prepared using an aseptic technique. A right paracostal incision was made starting immediately caudal to the 13th rib at the ventral margin of the epaxial muscles and continued cranioventrally along the ventral margin of each rib until an approximately 6‐8 cm incision was created. The external abdominal oblique, internal abdominal oblique, and transversus abdominis muscles were transected along the same line of exposure and retracted using a self‐retaining retractor. Hemostasis was achieved with bipolar cautery.

The caudate liver lobe was identified cranioventral to the right kidney and dorsal to the stomach. The vascular pedicle was ligated using either Surgitie ligating loop (Polysorb; Covidien, Mansfield, Massachusetts), vascular clips (Hemoclips®, Teleflex, Wayne, Pennsylvania), or encircling ligatures using 2‐0 silk suture. The liver lobe was removed distal to the ligated vessels. The pedicle was examined for bleeding prior to closure. The abdominal muscles were closed in 1 or 2 layers using polyglyconate suture. The subcutaneous tissue was closed with 4‐0 glycomer 631 in a simple continuous pattern. The skin was closed with either simple interrupted skin sutures or intradermal suture pattern.

#### 2.3.2 Ventral midline approach

The patient was placed in dorsal recumbency, and the abdomen was surgically clipped from cranial to the xyphoid to caudal to the pubis. The area was prepared using aseptic technique. A ventral midline incision was made from the xyphoid extending caudally. A reverse stab incision through the linea alba was made to enter the abdomen, and the incision was extended cranially and caudally using Metzenbaum scissors. Hemorrhage was controlled throughout the procedure using bipolar electrocautery.

The GI tract was retracted caudally to access the caudate liver lobe. The torsed caudate lobe was isolated, and the vascular pedicle was ligated with either Surgitie ligating loop (Polysorb), vascular clips (Hemoclips), or encircling ligatures using 2‐0 silk suture. The liver lobe was removed distal to the ligatures. The stump was inspected for bleeding prior to closure. The linea alba was closed using 3‐0 polyglyconate in a simple continuous pattern. The subcutaneous tissue was closed with 4‐0 glycomer 631 in a simple continuous pattern. The skin was closed with either simple interrupted skin sutures or an intradermal suture pattern.

### 2.4 Data analysis

Statistical analyses were conducted using SAS software (SAS Institute Inc., Cary NC), version 9.4. All reported *P*‐values are 2‐sided and *P* < .05 was used to define statistical significance. Descriptive statistics such as median and interquartile range were calculated for continuous variables and count and frequency were generated for categorical variables. The nonparametric Wilcoxon rank sum test was used in the surgical approach comparison for continuous outcomes because the data are not normally distributed, and Fisher's exact test was used for categorical outcomes.

## 3 RESULTS

### 3.1 Cadaver study

Upon entering the abdomen through a paracostal approach, the caudate liver lobe was readily identified in all 4 rabbits. In the first cadaver, a dorsoventral incision was made caudal to the last rib, and the cranial extent of the caudate liver lobe was difficult to access. The incision was then extended cranially along the ventral rib margin, creating excellent exposure of the caudate lobe hilus.

**FIGURE 1:**
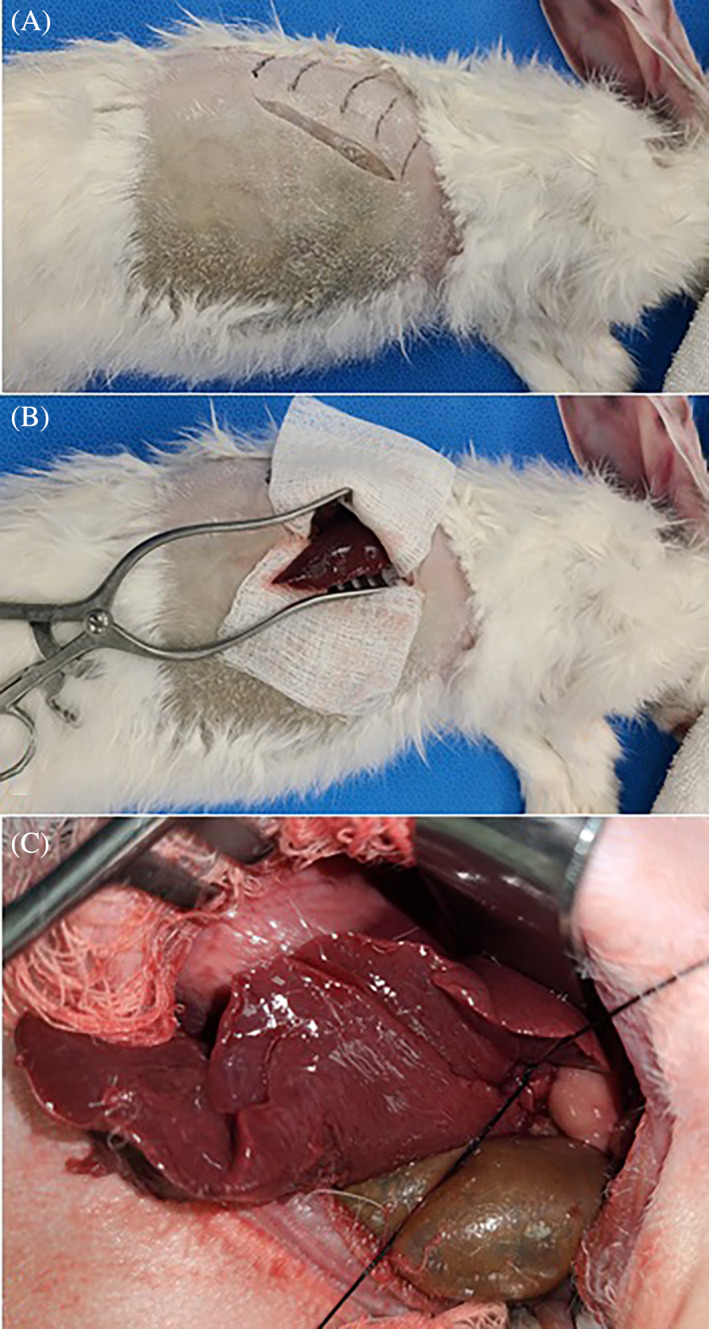
Paracostal approach to the caudate liver lobe. For all images, cranial is to the right, and dorsal is at the top of the image. (A) A linear to curvilinear incision is made caudal to the 13th rib at the level of the epaxial muscles. The incision is continued cranioventrally along the costal margin of the last rib and the ventral border of the ribs cranially. (B) The external abdominal oblique, internal abdominal oblique and transversus abdominus muscles are incised to match the skin incision, and the abdomen is entered. The abdominal muscles are retracted. The caudate liver lobe is readily identified. (C) The hilus of the caudate liver lobe is ligated with 3‐0 silk

**FIGURE 2:**
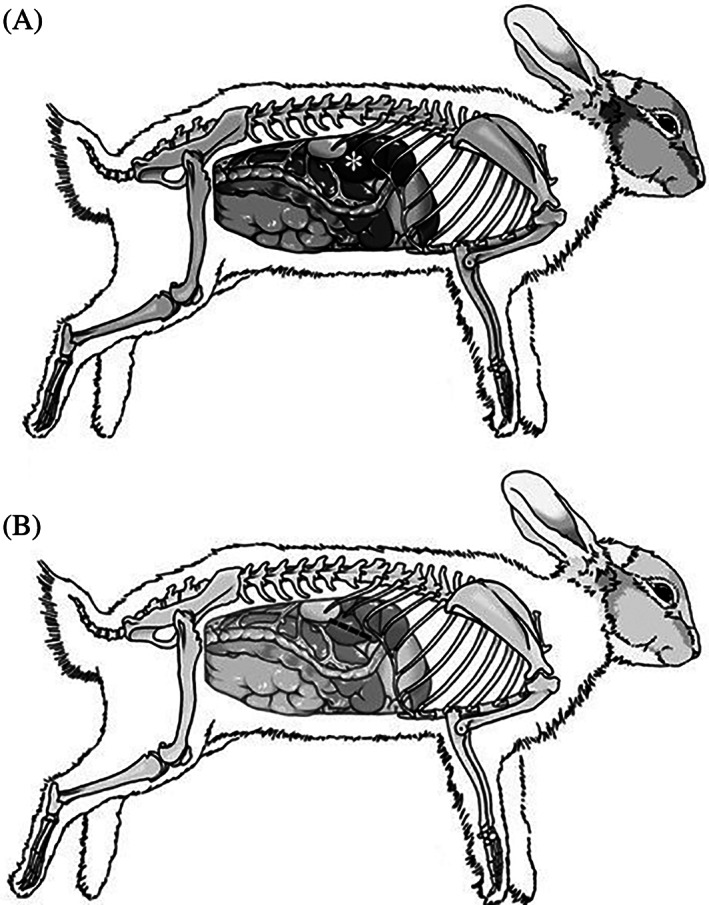
Illustration of (A) caudate liver lobe (*) in relation to the surrounding abdominal organs and rib cage. (B) paracostal incision (dashed line)

In the 3 remaining cadavers, the incision was curvilinear and followed the ventral edge of the ribs as described above (Figure 1). Incision size was approximately 6‐7 cm in all rabbits; however, statistical analysis was not performed due to the small sample size and variation to be expected with different breeds. Slight cranial retraction of the body wall was necessary to access the vascular pedicle of the liver lobe. The GI tract was ventral to the abdominal incision and did not need to be retracted to perform the lobectomy. Illustrations were then created to show the normal rabbit abdominal anatomy and the incision relative to the rabbit rib cage (Figure 2).

A single rabbit was used to perform a ventral midline approach to the caudate liver lobe for anatomical comparison. With the ventral approach, the stomach was overlying the caudate liver lobe and was retracted caudolaterally to access the lobe and vascular pedicle.

### 3.2 Clinical cases

Twenty‐three rabbits underwent liver lobectomy during the study period; however, 1 rabbit had a left lateral LLT and was excluded from the study. Twenty‐two rabbits met the inclusion criteria for the study. The median age at presentation was 4 years (range 1‐8 years). Fifteen males (10 castrated) and 7 females (6 spayed) were included. Breeds included New Zealand (n = 4), Holland Lop (n = 3), Rex (n = 3), and 2 each of Dutch, Flemish Giant, Lionhead, Mini Lop, French Lop, and mixed breed.

#### 3.2.1 Clinical signs

The most common clinical signs included anorexia (n = 19), lethargy (n = 14), decreased defecation (n = 13), abdominal distension or discomfort (n = 4), hiding (n = 2), or fever (n = 2).

#### 3.2.2 Diagnostic test findings

A packed cell volume (PCV), total solids (TS), and serum biochemical panel was submitted for 21 out of 22 patients (Table 1). Of these, 14 were found to be anemic. The median packed‐cell volume was 28% (range 15‐34%; reference range 30‐50%^24^). Elevation in alanine aminotransferase (ALT) was present in all patients. The median ALT was 497 IU/L and ranged from 155‐2250 IU/L (reference interval 45‐80 IU/L^25^). Only 3 rabbits had an elevated alkaline phosphatase (ALP). The median ALP was 54 IU/L and ranged from 12 to 229 IU/L (reference interval: 12 to 96 IU/L^25^).

**TABLE 1:** Preoperative physical examination and biochemical and hematologic values reported by group as median (interquartile range). Alanine transaminase was elevated in all rabbits in this study. There were no significant differences in preoperative variables between groups

	Ventral midline approach (n = 13)	Paracostal approach (n = 9)	*P*
Physical exam			
Weight (kg)	2.9 (2.7–4.4)	2.4 (2–4)	.1165
Temperature (F)	101.1 (100.3–103.4)	102.4 (101.2–102.5)	.7034
Heart rate	275 (248–280)	220 (210–240)	.0259
Biochemical			
ALT	549 (434–873)	446.5 (366–610.5)	.2937
ALP	54 (42–79)	61 (40.5–73)	1.0000
Hematologic			
PCV	27.5 (24.5–29)	28 (25–31)	.5668

Abbreviations: ALT, alanine transaminase; ALP, alkaline phosphatase, PCV, packed cell volume.

**FIGURE 3:**
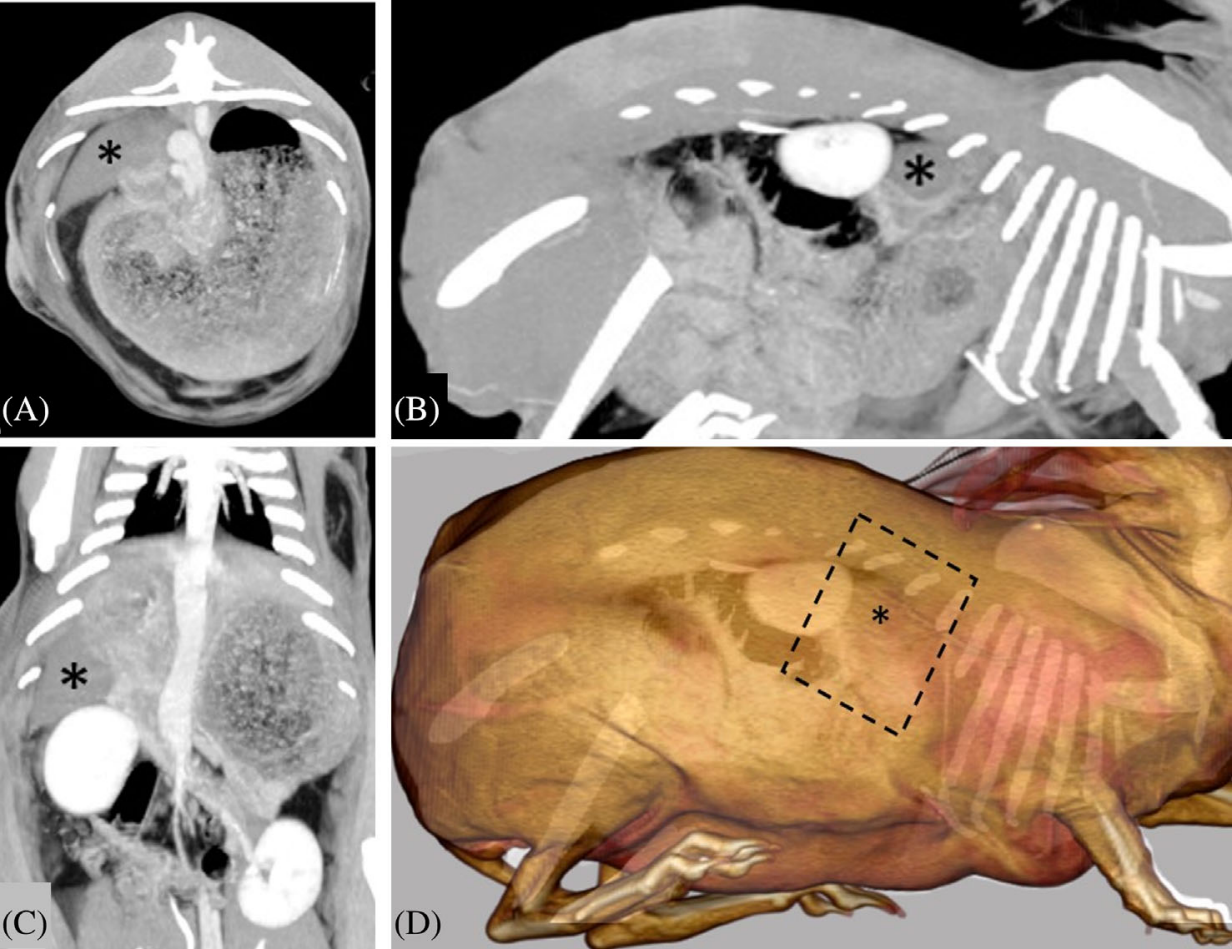
Preoperative postcontrast CT maximum intensity projections of the rabbit abdomen in (A) axial, (B) sagittal, and (C) dorsal planes, and (D) 3‐dimensional reconstruction. (*) denotes torsion of caudate liver lobe. The caudate liver lobe is mildly enlarged and hypoattenuating compared to the rest of the liver. There is minimal to no contrast enhancement of the hepatic parenchyma and no enhancement of the associated intrahepatic portal veins

Caudate LLT was diagnosed via abdominal ultrasound (n = 6), abdominal CT (n = 14) or a combination of ultrasound and CT (n = 2). Typical ultrasonographic findings included an enlarged, hypoechoic, heterogeneous caudate lobe with evidence of regional peritoneal effusion. Typical CT findings included an enlarged, rounded, hypoattenuating, heterogeneous, and non‐ to minimal contrast enhancement and a lack of contrast enhancing vasculature within the torsed caudate liver lobe (Figure 3).

An emergency liver lobectomy was performed in all 22 rabbits. The standard ventral midline approach was performed in 13 patients (Group A), and the paracostal approach was performed in 9 rabbits (Group B). Liver lobectomy was performed using the ventral midline or paracostal approach primarily based on surgeon preference and the results of diagnostic imaging. In 1 rabbit, the torsed caudate liver lobe was located more cranial and enclosed by the rib cage on CT images, and the attending surgeon elected a ventral midline approach to avoid obstruction by the ribs. There was no difference in age, sex, or ASA score between groups. Patients in Group A had a higher heart rate on presentation than Group B (*P* = .0259); however, the median heart rate for both groups was within normal limits (Table 2).

**TABLE 2:** Summary of perioperative variables reported by group as medians (interquartile ranges) except mortality and blood transfusion, which were reported as number of patients (percentages). Mortality was lower (*P* = .053) and postoperative time until eating was shorter (*P* = .0238) for patients undergoing the paracostal approach. There was no difference in anesthesia time, surgical time, need for transfusion, or hospitalization time between groups

Perioperative variables	Ventral midline approach (*n* = 13)	Paracostal approach (*n* = 9)	*P*
Anesthesia time (min)	75 (65–95)	95 (90–100)	.1397
Surgical time (min)	50 (35–55)	45 (40–55)	.9462
Blood transfusion^a^	3 (23)	0 (0)	.2403
Hospitalization duration^b^ (days)	2 (1–2)	2 (1–2)	.8324
Time to eating^b^ (h)	12 (12–24)	4 (3–5)	.0238
Mortality	5 (38)	0 (0)	.0537

^a^ Includes intraoperative and postoperative lapine whole‐blood transfusions.

^b^ Data are censored for patients that did not survive to discharge.

In Group A, the median anesthetic time was 75 minutes (range 35‐110 minutes). The median surgical time was 50 minutes (range 25‐65 minutes). The caudate liver lobe was removed successfully in 12/13 patients. The stomach was typically distended with gas and/or food material, requiring caudolateral retraction to access the torsed liver lobe. Intraoperatively, significant hemorrhage was noted in 2 patients and required blood transfusion. In 1 of these patients, the stomach was severely distended with gas and food material, inhibiting access to the vascular pedicle of the liver lobe. The pedicle tore during attempted ligation, resulting in severe hemorrhage and ultimately death. An additional 4 patients died following cardiac arrest before discharge from the hospital. Overall mortality for Group A was 5 of 13 patients (38.5%).

In Group B, the median anesthesia time was 95 minutes (range 80‐100 minutes). The median surgical time was 45 minutes (range 30‐60 minutes). The caudate liver lobe was successfully removed in all 9 patients without the need to convert to a ventral approach. No intraoperative complications were reported in any patients from Group B. Postoperative complications were reported in 2 patients from Group B. One patient exhibited significant respiratory distress approximately 5 hours after surgery. It was treated with oxygen supplementation and had normal respiratory rate and effort within an hour. This patient was discharged from the hospital the following day. An additional patient had waxing and waning hyperthermia (>103.0F) for 3 days after surgery but was eating and defecating normally throughout her hospitalization. All patients from Group B recovered and were discharged from the hospital.

There was no difference in total anesthesia time (*P* = 0.1397) or surgical time (*P* = 0.9462) between groups. Mortality rate gave a *P* value of 0.053. Receiving a blood transfusion was not different between groups (*P* = 0.2403).

Median time to eating after surgery was recorded in 17 of 22 patients. Time to eating was significantly lower for Group B (4 hours) compared to Group A (18 hours) (*P* = .0162). Time to eating remained lower for patients in Group B when patients that died were censored (*P* = .0238). All patients in Group B started eating voluntarily within 24 hours of surgery.

The median postoperative hospitalization time was 2 days for both groups and was not statistically significant.

An effort was made to compare the surgeon performing the procedure in both groups; however, given the large number of surgeons and residents within hospital, statistical comparison was not possible due to small sample size for each surgeon. When visibly assessing the distributions of surgeons and residents between the 2 approaches, there were no obvious differences that warranted concern.

## 4 DISCUSSION

This is the largest study of rabbit caudate LLT treated by emergency liver lobectomy reported. Overall survival with surgery was 77.2% (17/22), with 100% survival when the liver lobe was removed through a novel right paracostal approach (9/9). This approach provided excellent exposure of the caudate liver lobe, with similar anesthetic and surgical times to rabbits treated via a ventral midline approach.

Medical management has previously been studied as an alternative to surgical management for rabbits with LLT with a reported survival rate of 43%.^2^ Early case reports of liver lobectomy in rabbits documented inconsistent mortality rates (0‐100%).^6^, ^8^, ^14^ A more recent retrospective study that compared medical and surgical management of LLT reported 100% survival for patients treated by liver lobectomy.^2^, ^8^ All previous reports of liver lobectomy in rabbits described surgery through a ventral midline approach.^8^, ^14^ It is unclear from these studies whether liver lobectomy was recommended for all rabbits presenting with LLT or only for a subset of patients deemed safe to anesthetize. In the current study, all rabbits underwent surgical treatment for LLT regardless of ASA status given the poor survival reported with supportive care alone. The authors of this study recommend medical management as an option only when surgery is cost‐prohibitive for the client.

The difference in mortality between groups in this study did not reach significance (*P* = 0.0537); however, a greater difference might be achieved with larger case numbers. Without a difference in surgical or anesthesia times between groups, there is likely an alternative explanation for improved survival with the paracostal approach.

One possible explanation for increased mortality associated with the ventral midline approach is the placement of patients in dorsal recumbency for anesthesia and surgery. Anecdotally, rabbits placed in this position have a more challenging anesthesia experience secondary to the Pir distended GI tract compressing the vena cava and diaphragm and the need for greater assistance with ventilation. Despite commentary from our anesthesiologists and certified veterinary technicians that rabbits in lateral recumbency have a better anesthesia experience, we were unable to find objective data in our anesthesia records that would explain differences in outcome. Varying methods of monitoring blood pressure, lack of controlled perianesthetic drugs, and variability in continuous parameter assessment likely all contribute to the challenge of comparing anesthetic events among groups.

Another possible explanation for improved outcome with a paracostal approach is improved exposure of the caudate liver lobe vascular pedicle. A small amount of evidence to support this fact in this study was the single rabbit that bled fatally during surgery in the ventral midline group. Significant efforts were made to reach the bleeding pedicle; however, the distended stomach obstructed visualization and access. Despite decompression of the stomach and a blood transfusion, the patient did not regain consciousness after surgery. The paracostal approach allowed for easy ligature placement around the hilus and the ability to carefully inspect the vascular pedicle after lobectomy without obstruction by the GI tract.

An unanticipated benefit of the paracostal approach was the rapid return to eating after surgery. Rabbits diagnosed with LLT commonly have concurrent GI stasis. Gastrointestinal stasis can result in gas distended loops of intestine being encountered during surgery,^2^, ^26^ which then require retraction to improve exposure to the caudate liver lobe hilus. A rapid return to eating after surgery is important to prevent postoperative ileus in rabbit patients. Postoperative ileus can lead to pain, decreased appetite and if left untreated, can result in death within days of anesthesia.^27^ The paracostal approach circumvented the need for GI retraction, which may likely have contributed to improved appetite and survival after surgery.

The rabbit GI tract is very intolerant to manipulation to the extent that rabbits are frequently used as models for postoperative abdominal adhesion formation. Methods to avoid handling of the GI tract are recommended to prevent this complication.^22^ Because we did not follow the outcome in the rabbits in this study beyond the short‐term perioperative period, we do not know if the paracostal approach minimizes adhesion formation in the future. Additional studies would be recommended to compare the long‐term outcome of the different approaches.

It is important to recognize that all rabbits in this study underwent advanced imaging (abdominal ultrasound and/or CT) prior to surgery. Imaging is critical to differentiate LLT from other diseases in rabbits that cause nonspecific clinical signs (eg inappetence, lethargy, and hiding) and elevated liver enzymes such as hepatic toxicity and metabolic or infectious disease.^28^ In a previous study from our hospital, CT was found to be highly sensitive and specific for LLT in rabbits and required only injectable sedation to perform. Computed tomography required similar or even less time than abdominal ultrasound to reach a diagnosis.^23^

A critical finding from the cadaver study that was importance of close proximity of the paracostal incision to the rib cage. A linear dorsoventral incision limited cranial exposure of the caudate liver lobe, whereas a curvilinear incision along the ventral edge of the ribs maximized exposure. Further, placement of the incision in this orientation allows the surgeon to extend the incision as far cranial as the xiphoid if necessary. One might be concerned about the possible additional time required to close the abdominal wall muscle layers; however, the median surgical time was no different between groups.

A limitation of the paracostal approach is the inability to access the remainder of the liver. This is an important consideration when determining the best approach to liver lobectomy for an individual case. There were 23 rabbits treated surgically for LLT during the timeframe of this study; however, 1 rabbit had a left lateral LLT which was removed through a ventral midline approach and was not included in this study. Selection of approach, even when the torsed lobe is known to be the caudate lobe, should be based on careful analysis of diagnostic imaging with particular attention to where the caudate lobe lies relative to the rib cage. The approach was not randomized in this study for 2 reasons. One reason was clinician preference, as some clinicians were reluctant to try the new approach until proven advantageous. The other reason was based on individual anatomy of the presenting rabbits. There was a case, treated by the author, in which the caudate liver lobe was deemed too cranial relative to the thoracic wall to be removed through a paracostal approach. The ventral midline approach was elected in this case.

Limitations of this study include the retrospective nature of data analysis, limited case numbers, and lack of control for anesthesia protocols or attending surgeon and anesthetist. Sample size was too small to analyze the effect of the individual surgeon on outcome; however, there were no obvious differences in distribution of surgeons and residents performing the 2 different approaches.

Despite these limitations, the paracostal approach allowed excellent exposure of the caudate liver lobe in rabbits while avoiding excessive GI tract manipulation. Rabbits treated through a paracostal approach ate earlier after surgery and had an improved rate of survival. Future studies are necessary to determine if there is a long‐term beneficial effect of treating caudate LLT in rabbits through a paracostal approach.

## AUTHOR CONTRIBUTIONS

Leonard KC, DVM: Design of the study, acquisition of data by performing surgery, analysis of data, and manuscript preparation. Zhao Q, MS (statistician): Analysis and interpretation of data, manuscript approval. Taber RH, BS: Acquisition of data and manuscript approval. Colopy SA, DVM, PhD, DACVS: Conception and design of the study, acquisition of data by performing surgery, analysis of data, and manuscript preparation.

## CONFLICT OF INTEREST

The authors declare no conflicts of interest related to this report.
